# Acute Effects of Slow, Moderate and Fast Tempo Dynamic Stretching Exercises on Power in Well-Trained Male Wrestlers

**DOI:** 10.5114/jhk/183543

**Published:** 2024-05-17

**Authors:** Cem Kurt, Gökhan Tuna, İmren Kurtdere

**Affiliations:** 1 Kirkpinar School of Sport Sciences, Division of Movement and Training Sciences, Trakya University, Edirne, Turkey; 2 Institute of Graduate Studies, Division of Movement and Training Sciences, Istanbul University-Cerrahpaşa, Istanbul, Turkey

**Keywords:** velocity, repeated jump test, wrestling, performance

## Abstract

Due to the potential detrimental effects of static stretching exercises on subsequent muscle power performance, athletes and trainers have started to replace static stretching with dynamic stretching exercises in their training routines. However, there are no well-accepted guidelines regarding dynamic stretching variables, including tempo/velocity, volume (reps and sets), etc. Therefore, this study aimed to evaluate the acute effects of slow, moderate, and fast tempo dynamic stretching exercises on jump height, relative power, the reactive strength index, and leg stiffness in well-trained male wrestlers. Seventeen wrestlers (aged 20.00 ± 4.06 years, wrestling experience 6.00 ± 3.09 years, and training volume per week 10.00 ± 5.69 hours) took part in the experiment under four conditions (control session, slow tempo dynamic stretching, moderate tempo dynamic stretching, and fast tempo dynamic stretching) on separate days with a 72-h interval in between, following a randomized, crossover study design. The control session consisted of a 10-min jog on a motor-driven treadmill at 6 km/h and a 0% slope. Dynamic stretching sessions consisted of seven dynamic stretching exercises performed at 50 bpm, 100 bpm, and 120 bpm, following a 5-min warm-up on a treadmill at 6 km/h and a 0% slope. After each condition, wrestlers performed a 2 x 30-s repeated vertical jump test with 5-min rest intervals in between. The best results for jump height, relative power, the reactive strength index, and leg stiffness were registered for statistical analysis. One-way repeated ANOVA results demonstrated that there were no significant differences in pairwise comparisons of all variables obtained after the four different conditions (p > 0.05). Overall, none of the slow, moderate, and fast tempo dynamic stretching exercises led to a change in repeated jump performance of well-trained male athletes. Further studies are needed to clarify the acute effects of different tempo dynamic stretching on muscular performance in well-trained wrestlers.

## Introduction

It is well known that athletes generally perform a warm-up before a match or training to increase performance and decrease the incidence of sports-related injuries. A traditional warm-up consists of a light aerobic exercise (jogging, cycling, etc.) and subsequent stretching exercises, such as static stretching (SS), dynamic stretching (DS), ballistic stretching (BS), and proprioceptive neuromuscular facilitation (PNF), either alone or in combination (Behm and Chaouachi, 2011). SS exercises require holding a muscle in a stretched position for a period of time to reach the threshold of the muscle. Yet, some authors have argued that stretching a muscle for longer than 90 s (e.g., 3 sets of 30 s) may lead to a decrement in explosive muscle performance (e.g., sprinting, jumping, agility, and change of direction) due to tendon slack and autogenic inhibition (Akınoğlu et al., 2021; Avloniti et al., 2016; Behm and Chaouachi, 2011; Erol et al., 2023).

Therefore, athletes have started replacing SS exercises with DS exercises due to the possible negative effects of SS on muscle performance. While Behm and Chaouachi (2011) reported that DS activities would either have no detrimental effect or may augment performance, Zmijewski et al. (2020) reported that DS improved repeated-sprint performance to a greater extent than static stretching and no stretching in female handball players. Araya-Ibacache et al. (2022) also suggested incorporating dynamic stretching exercises instead of static stretching before a competition in female volleyball players, since static stretching produced a decrease in explosive strength, whereas dynamic stretching increased this variable. DS refers to controlled movements that occur rhythmically and at a certain tempo throughout the active range of motion of each joint (Beedle et al., 2008). DS is a type of stretching that involves a few sets of movements, each lasting 10–20 s, with a tempo ranging from 50 to 120 beats per minute (Opplert and Babault, 2018). It has been accepted that DS applied during the warm-up is more effective than SS in terms of muscle performance improvement due to several physiological mechanisms. These mechanisms include increased intramuscular temperature, decreased muscle viscosity (via reducing inter-joint resistance), enhanced reflex sensitivity, summation and synchronization of motor units, an increased muscle blood flow, and post-activation potentiation (PAP) (Opplert and Babault, 2018).

Fletcher and Anness (2007) concluded that active DS was more effective than passive SS or a combination of passive SS with active DS in improving 50-m sprint performance in experienced sprinters. In that study, DS exercises were specifically chosen to replicate various phases of the sprinting cycle and target the key lower body muscles utilized in sprinting, including the gastrocnemius, gluteals, hamstrings, quadriceps, and hip flexors. Those exercises, namely straight leg skipping, walking high knees, skipping high knees, running high knees, and flick backs, were performed in a repeated manner over a 20-m distance, followed by a recovery period involving walking back to the starting point. Performance enhancement following DS was attributed to the practice of precise movement patterns (Fletcher and Anness, 2007). This aids in improving proprioception and preactivation, thereby facilitating a seamless transition from eccentric to concentric muscle contractions, a crucial aspect for generating high running speeds (Fletcher and Jones, 2004).

In another study conducted by Jaggers et al. (2008), a comparison was made between DS and BS exercises with regard to their impact on vertical jump height, force, and power in college students. In that study, participants engaged in two sets of five DS exercises, each exercise being performed five times. The execution involved a gradual, slow-paced approach at the beginning, followed by ten repetitions executed as rapidly and forcefully as possible, all performed without any bouncing. This led to a total of 15 repetitions per exercise. Concerning BS exercises, participants engaged in rapid bouncing for 30 s, maintaining a rhythm of 126 beats per minute, regulated by a metronome. Similarly to the DS session, each ballistic exercise was conducted in two sets. Notably, in the study conducted by Jaggers et al. (2008), it was reported that neither DS nor BS resulted in an increase in vertical jump or force performance.

Even though DS is recommended to athletes for decreasing sport-related injuries and performance improvement instead of SS, there is no consensus among researchers on a well-accepted guideline regarding the velocity of DS, DS duration, volume of DS exercises, etc. (Behm et al., 2016). In the absence of such specific DS exercise guidelines, Yamaguchi and Ishii (2014) argued that optimal protocols of DS should be performed “as fast as possible” and consist of “10–15 repetitions” or “10 yards to 20 meters” in distance x “1–2” sets.

As far as we understand, most studies have evaluated the effectiveness of the DS volume rather than DS velocity (Costa et al., 2013; Dallas et al., 2019; Ryan et al., 2014; Turki et al., 2011). Therefore, we aimed to evaluate the effectiveness of DS exercises performed at tempos of 50 b/min, 100 b/min and 120 b/min on power including maximal jump height (JH_max_), minimum jump height (JH_min_), mean jump high (JH_mean_), relative power (R-PPO), the reactive strength index (RSI) and leg stiffness (K_leg_) in male wrestlers. The first hypothesis of the study was that as the velocity of DS increased, power performance would also increase. We also hypothesized that a fast tempo in dynamic stretching would lead to a greater increase in the heart rate (HR) and body temperature.

## Methods

### Participants

Seventeen male wrestlers were recruited for the study. Descriptive characteristics of participants are presented in Table 1. Prior to the start of the study, all the athletes were fully informed about the study protocols and risks associated with participation. Written informed consent was obtained from all the athletes. The study was carried out in accordance with the Helsinki Declaration and was approved by the Ethics Committee of the Trakya University Medical Faculty (approval code: TÜTF-GOBAEK 2022/80; approval date: 07 March 2022). Inclusion criteria were: a) absence of musculoskeletal injuries for at least six months before the study; b) age of 18 years or above; c) at least three years of experience in the sport; d) a sports license for the 2021–2022 season; and e) active participation in sports training (≥4–6 times per week). Exclusion criteria were: a) reporting usage of any ergogenic supplements, such as creatine, amino acids, and protein powder, b) a history of orthopedic problems, such as hamstring-quadriceps injuries, fractures, surgery or pain in the spine or hamstring-quadriceps muscle over the past 6 months. The effect size (*d* = 0.62) and other sample size determination variables (α = 0.05 and 1-β = 0.80) were obtained from the study by Fletcher (2010). The calculation, when entered into the G*Power 3.1.9.2 program, showed that 13 participants would be sufficient for our study.

**Table 1:** Characteristics of study participants.

Variables	M ± SD
Age (years)	20.0 ± 4.06
Bodu height (cm)	1.75 ± 0.08
Body mass (kg)	79.0 ± 10.92
Exercise Experience (year)	6.0 ± 3.09
Training volume per week (hours)	10.0 ± 5.69

M ± SD: Mean ± Standard Deviation

### Procedures

This study was conducted between April and July 2023 at the Human Performance Laboratory of the Kirkpinar School of Sport Sciences. All trials were performed at the same time of the day (5.00 p.m.–7.00 p.m.) to avoid any effect of circadian variations on the study results. The indoor temperature was maintained at 20–21°C throughout all experimental sessions.

### Devices

The laboratory temperature was measured using a digital thermohygrometer (Eurofrost model BT-2, PRC). The wrestlers’ heart rate was measured using a telemetry heart monitor RS 400 (Polar Electro Oy, Professorintie 5, FI-90440, Kempele, Finland). The wrestlers’ body temperature was measured using an infrared thermometer (Quadro LK001) on their foreheads.

The body mass and height of wrestlers were measured using a digital scale (Seca 769, Turkey). Body mass and height were measured while participants were barefoot and wearing short tights and short-sleeved shirts.

Muscular power: Power of wrestlers including maximal jump height (JH_max_), minimum jump height (JH_min_), mean jump height (JH_mean_), relative power (R-PPO), the reactive strength index (RSI) and leg stiffness (K_leg_) were evaluated using a 30-s repeated vertical jump test (30-s RVJ). R-PPO represents power/body mass. The test protocol consisted of a 2 x 30-s RVJ test with 5-min rest intervals in between (Karuk et al., 2022). Measurements were taken using an electronic jumping platform (Smart Speed, Fusion Sport, Qld, Australia). The SmartJump contact mat and also the 30-s RVJ test have been considered reliable and valid for evaluating anaerobic performance variables including JH_max_, JH_mean_, etc., as well as ground contact time and vertical stiffness (ICC ranging from 0.94 to 0.98) (Dal Pupo et al., 2013, 2014; Reeve and Tyler, 2013). The RVJ was performed at a starting knee angle of 100°, which was held for 1 s, with no countermovement. It was initiated from a standing position, then quickly lowering to a 100° knee angle and rebounding to a maximal jump height (McCaulley et al., 2007). All wrestlers were instructed to put their hands on their waists and perform continuous vertical jumps at maximal strength with no pause between jumps during tests (Hespanhol et al., 2006). Wrestlers were asked to remain with their trunk in the vertical position with no excessive forward movement, and having their knees extended during the flight phase, in order to avoid influencing the results (Hespanhol et al., 2006). During the test, researchers verbally motivated participants to exert maximum effort (Karuk et al., 2022)

### Exercise Protocols

Before each session, wrestlers lay in a supine position for 15 min. At the end of this time period, their resting heart rate and forehead temperature were recorded. The wrestlers’ heart rate and forehead temperature were also recorded right after each dynamic stretching exercise session. Wrestlers participated in four different exercise and test sessions, which were called a control session, a slow tempo dynamic stretching session, a moderate tempo dynamic stretching session, and a fast tempo dynamic stretching session. We tried to design a relatively equal length of time for the three different velocities of DS protocols. These sessions were conducted with a 72-hour rest interval in between following a randomized crossover study design.

Warm-up: Each session started with a five-minute run on a motor-driven treadmill (SportsArt T630, USA) at 6 km/h and a 0% slope.

Control session: This session consisted of a 10-min run at 6 km/h and a 0% slope on a motor-driven treadmill as in the warm-up. Afterwards, a 2-min rest interval was given to wrestlers. At the end of the 2-min rest interval, they performed a 2 x 30-s RVJ test with 5-min rest intervals.

Dynamic stretching exercises: Wrestlers participated in one of the DS sessions consisting of tempos of 50 b/min, 100 b/min or 120 b/min according to block randomization. The tempo of the exercises was controlled by a digital metronome. Each DS session consisted of seven exercises including a squat jump, a straight leg march, high knee skipping, running butt kicks, walking lunges, heidens, and vertical ankle jumps. Each exercise involved 2 x 10 repetitions with a 10-s rest interval between sets and a 30-s rest interval between exercises.

### Statistical Analysis

Data are presented as mean and standard deviation. The Shapiro-Wilks test was used to check for normality, and it was found that normality assumptions were met. A paired *t*-test was performed for matched pairs of heart rates (before vs. after sessions). Cohen’s *d* was used to assess effect size and categorized as no effect (0–0.2), small effect (0.2–0.5), medium effect (0.5–0.8), and large effect (> 0.8). To assess differences between dynamic warm-up effects (control, 50 bpm, 100 bpm, 120 bpm) in the different sessions, one-way repeated-measures analysis of variance (ANOVA) was used. Greenhouse-Geisser adjustments of the *p*-values were reported since the sphericity assumption was violated (*p* < 0.05). Partial eta squared ($\eta_{p}^{2}$) was used to determine effect size and categorized as small (0.01–0.06), medium (0.06–0.14) and large effect (> 0.14). The Bonferroni adjustment was used for multiple comparisons. Data analysis was performed using SPSS software (IBM SPSS Statistics for Windows, Version 25.0. Armonk, NY: IBM Corp). The level of significance was set at *p* ≤ 0.05.

## Results

ANOVA results of JH_max_, JH_min_, JH_mean_, R-PPO, RSI and K_leg_ are presented in Table 2. The results of one-way repeated ANOVA for the heart rate and body temperature between different sessions are shown in Table 3. The heart rate and body temperature were measured before and after each session and the difference within each session is shown in Table 4.

**Table 2:** ANOVA results of studied variables.

Parameters	Session	M ± SD	*F*_(3,48)_	*p*	$\eta_{p}^{2}$
JH_max_	Control	33.319 ± 8.07	0.696	0.559	0.042
	First	31.589 ± 3.954			
	Second	32.678 ± 7.485			
	Third	31.341 ± 5.490			
JH_min_	Control	17.126 ± 7.632	0.737	0.460	0.044
	First	16.520 ± 7.206			
	Second	18.294 ± 5.292			
	Third	15.801 ± 7.331			
JH_mean_	Control	24.634 ± 4.995	0.038	0.953	0.002
	First	24.628 ± 4.676			
	Second	24.954 ± 4.433			
	Third	24.717 ± 5.460			
R-PPO	Control	44.898 ± 7.047	0.800	0.454	0.048
	First	43.596 ± 2.962			
	Second	45.442 ± 9.124			
	Third	43.497 ± 4.096			
RSI	Control	0.735 ± 0.234	2.530	0.104	0.137
	First	0.653 ± 0.241			
	Second	0.851 ± 0.490			
	Third	0.663 ± 0.106			
K_leg_	Control	11.914 ± 8.034	2.789	0.085	0.149
	First	8.258 ± 5.021			
	Second	8.546 ± 3.844			
	Third	10.055 ± 5.100			

R-PPO: Relative power; RSI: Relative Strength Index; Max: Maximal; Min: Minimum; K_leg_: Leg stiffness; M ± SD: Mean ± Standard Deviation; $\eta_{p}^{2}$: Partial eta squared

**Table 3:** The results of one-way repeated ANOVA for the heart rate and body temperature between particular sessions.

Variables	Session	M±SD	*F*_(3,48)_	*p*	$\eta_{p}^{2}$
Before Session HR	Control	76.76 ± 8.18	0.199	0.896	0.012
	First	75.35 ± .7.72			
	Second	76.71 ± 9.23			
	Third	75.35 ± 7.72			
After Session HR	Control	165.65 ± 8.41*	3.372	0.037	0.174
	First	166.29 ± 6.98*^†^			
	Second	170.71 ± 5.35			
	Third	171.53 ± 5.32*^†^			
Before Session Body Temp.	Control	36.31 ± 0.21	0.233	0.770	0.014
	First	36.31 ± 0.215			
	Second	36.33 ± 0.20			
	Third	36.36 ± 0.29			
After Session Body Temp.	Control	36.31 ± 0.21’’	2.184	0.116	0.120
	First	36.312 ± 0.215			
	Second	36.17 ± 0.30			
	Third	36.15 ± 0.28’’			

HR: Heart rate; M ± SD: Mean ± Standard Deviation; $\eta_{\rho }^{2}$

: Partial eta squared. * *p* = 0.033, significant difference between first and third sessions (mean difference = 5.882). ^†^ *p* = 0.035, significant difference between first and third sessions (mean difference = 5.235). ’’: *p* = 0.043, significant difference between first and third sessions (mean difference = 0.165).

**Table 4:** Paired T-test results of the heart rate and body temperature.

Variable	Session	Before vs. After	*t*_(16)_	*p*	Cohen’s *d*
Heart Rate (bpm)	Control	76.76 ± 8.18 vs. 165.65 ± 8.41	−37.212	< 0.001	9.025
	First	75.35 ± 7.72 vs. 166.29 ± 6.98	−41.267	< 0.001	10.00
	Second	76.71 ± 9.23 vs. 170.71 ± 5.35	−33.782	< 0.001	8.19
	Third	75.35 ± 7.72 vs. 171.53 ± 5.32	−49.033	< 0.001	11.89
Body Temperature (°C)	Control	36.31 ± 0.215 vs. 36.31 ± 0.21	0.00	1.0	0.0
	First	36.31 ± 0.215 vs. 36.31 ± 0.21	0.00	1.0	0.0
	Second	36.33 ± 0.20 vs. 36.31 ± 0.21	0.263	0.796	0.64
	Third	36.36 ± 0.29 vs. 36.15 ± 0.28	5.944	<0.001	1.44

**Figure 1:**
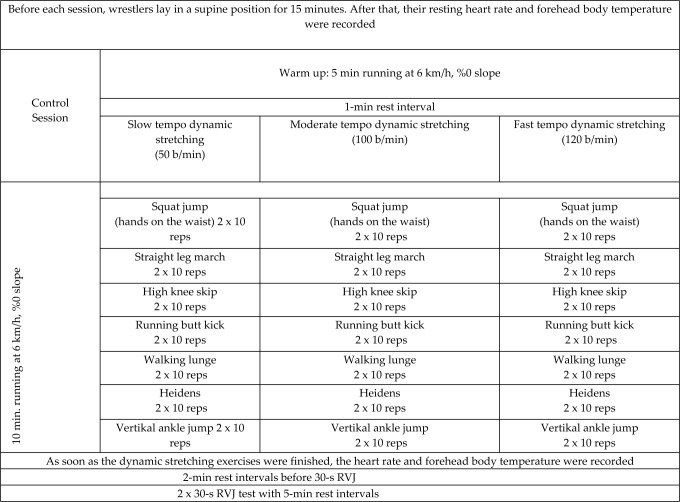
Flowchart of the study.

The results of one-way repeated ANOVA for variables between different sessions are shown in Table 2. There were no significant differences in pairwise comparisons of all variables (*p* > 0.05).

There were significant differences in heart rates taken before and after each session (before vs. after) at a level of < 0.001. In terms of effect size, the greatest differences were found after the third session (*d* = 11.89). Regarding body temperature, differences in values were observed only in the third session (before vs. after), with an effect size of *d* = 1.44 (Table 4).

## Discussion

This study aimed to evaluate the effectiveness of DS exercises performed at tempos of 50 b/min, 100 b/min and 120 b/min on power including JH, R-PPO, RSI and K_leg_ in well trained male wrestlers. The main finding of the study was that none of the DS tempos elicited an increase in JH, R-PPO, RSI, and K_leg_. Thus, the first hypothesis of the study (that increasing the velocity of dynamic stretching would increase power performance) could not be verified. The HR and body temperature increased after fast tempo DS to a greater extent compared to slow and moderate tempo DS (Cohen’s *d* values of 11.89 and 1.44 for the HR and body temperature, respectively; Table 4). Based on these results, we can conclude that the second hypothesis of the study (that fast tempo DS would lead to a greater increase in the heart rate and body temperature) was verified.

Muscular performance improvements after a single bout of DS are likely attributed to post activation potentiation (PAP) or post activation potentiation enhancement (PAPE) mechanisms (Fletcher, 2010; Opplert and Babault, 2018). Enhancements in muscle twitch properties and voluntary dynamic force production subsequent to an acute-short bout of high-intensity voluntary exercise are known as PAP (Bishop, 2003; Opplert and Babault, 2018). However, Blazevich and Babault (2019) have reported that as long as we do not evaluate the twitch force response of a muscle after heavy resistance exercise or explosive movement, we cannot explain performance enhancement through PAP. It has been argued that if there is no twitch force assessment, at this time performance enhancement after heavy resistance training or explosive manner exercise can be explained by PAPE (Blazevich and Babault, 2019; Opplert and Babault, 2018). PAPE is characterized by changes in: muscle temperature, muscle/cellular water content, the blood flow to the muscle, hormonal status, the heart rate, as well as increases in spinal reflex activity and in motor unit activation, and decreases in viscosity of the muscle (Blazevich and Babault, 2019; Opplert and Babault, 2018; Vargas-Molina et al., 2021). In this study, the heart rate and body temperature of wrestlers before and after each dynamic stretching exercise session were also recorded to be able to explain possible performance (JH_max_, JH_min_, JH_mean_, R-PPO, RSI and K_leg_) enhancement after DS exercises. The highest heart rate and body temperature were registered in the fast tempo dynamic stretching session (Table 4). This was not a surprise due to the fact that DS exercises were performed as fast as possible (120 b/min). Although significant differences were found between the resting heart rate and body temperature after the fast tempo dynamic stretching session, there were no performance changes in JH_max_, JH_min_, JH_mean_, R-PPO, RSI and K_leg_ to be explained through heart rate and body temperature increases.

In contrast to the results of our study, Fletcher (2010) reported that a leg swing applied at 100 b/min) was more effective than a leg swing applied at 50 b/min in terms of vertical jump (VJ) performance. The improvement in jump performance was explained by increasing muscle-tendon unit (MTU) stiffness, greater nervous system activation, and increases in the heart rate and core temperature (Fletcher, 2010).

Furthermore, Faigenbaum et al. (2006) noted that the intensity, volume, duration and the type of DS routines were critical variables that might potentially influence subsequent performance. Additionally, Hough et al. (2009), reported that seven minutes of DS when each exercise was performed five times slower and then 10 times as quickly as possible without bouncing increased VJ performance by enhancing the neuromuscular drive of the relevant muscles.

On the other hand, Ryan et al. (2014) suggested that DS routines lasting approximately 6–12 min, performed following a 5-min jog, resulted in similar increases in VJ performance and flexibility. Ryan et al. (2014) also argued that six minutes of DS of the hip and thigh musculature might be the most appropriate volume to improve VJ performance, flexibility, and muscular endurance in recreationally active men, and that longer duration of DS routines (up to 12 min) may impair repetitive high-intensity activities.

Opplert and Babault (2018) and Yamaguchi and Ishii (2014) reported that high volume or long-duration DS exercises had no detrimental effect on performance. However, Ryan et al. (2014) argued that when high volume or long-duration DS exercises were applied at high velocity (> 100 b/min), the cumulative effect might lead to performance decrement due to muscular fatigue instead of muscular potentiation improvement.

In our study, slow, moderate and fast tempo DS exercises were completed in 9.3 min, 7.4 min and 6.8 min including rest intervals between sets and exercises, respectively. Due to difficulties in suggesting an optimal protocol for DS, we cannot interpret whether DS sessions with high volume, a fast tempo or a combination of them affect performance. Specifically, the exercise tempo of 120 bpm performed in our study might be somewhat tiring and could potentially hinder performance enhancement. Furthermore, we noticed that certain athletes encountered difficulties in sustaining the fast tempo, and there appeared to be an inconsistency between the tempo and certain exercises during the fast tempo dynamic stretching session.

The inconsistent results between the literature and our study might be caused by several factors including gender, the performance level of study subjects, training and sports experience of athletes, and the intensity, volume, duration and the type of DS exercises (Christensen and Nordstrom, 2008; Faigenbaum et al., 2006; Fletcher and Anness, 2007). In addition to the factors mentioned above, selecting exercises tailored to specific sport disciplines could be an even more crucial factor. Some authors recommend that exercises to be chosen should be selected from the exercises used in the respective sports discipline and those utilized in athletes’ daily training routines (Fletcher and Anness, 2007; Holt and Lambourne, 2008).

In our study, DS exercises were selected from well-known and common exercises among the athletic population. However, these exercises may not all be suitable for wrestlers. This could represent a significant limitation of the study. Specifically, during fast tempo DS exercises, we observed that certain exercises, such as the “walking lunge”, were not suitable for performing as rapidly as required. For future research, exercises should be selected based on the specific sport discipline and the cadence of the metronome.

## Conclusions

None of the slow, moderate or fast tempo DS exercises resulted in a change in the repeated jump performance of well-trained male athletes. Although Fletcher (2010) proposed that a faster DS component (100 bpm) would better prepare an athlete for optimal performance, the findings of our study did not align with the Fletcher’s suggestion. Further research is necessary to clarify the immediate effects of DS at different tempos on muscular performance in well-trained wrestlers. This research should be designed to explore the acute effects of DS using varying tempos, sets, and repetition configurations.
